# Real‐Time and Non‐Invasive Detection of Respiratory Viral Infections Using an Intelligent Odor Monitoring System (IOMS)

**DOI:** 10.1002/advs.76244

**Published:** 2026-06-23

**Authors:** Yajie Shen, Weifeng Yuan, Long Li, Yucheng Zheng, Kenan Liu, Hegeng Li, Hua‐Yao Li, Yongliang Zhao, Binzhou Ying, Lanpeng Guo, Wenjian Zhang, Shu‐Ming Kuo, Zirui Zhang, Yufan Deng, Bohan Yin, Zhaocheng Luo, Ke Xu, Huan Liu

**Affiliations:** ^1^ State Key Laboratory of Virology and Biosafety Institute for Vaccine Research College of Life Sciences Wuhan University Wuhan Hubei P. R. China; ^2^ School of Integrated Circuits Wuhan National Laboratory for Optoelectronics Optics Valley Laboratory Huazhong University of Science and Technology Wuhan Hubei P. R. China; ^3^ School of Public Health, Wuhan University Wuhan Hubei P. R. China

**Keywords:** machine learning, respiratory infection, sensor array

## Abstract

Real‐time monitoring of infection‐associated volatile organic compounds (VOCs) offers a non‐invasive pathway for early respiratory infection detection. However, diagnostic precision remains challenged by complex VOC mixtures, low analyte abundance, and significant biological variability. This study introduces an Intelligent Odor Monitoring System (IOMS) guided by infection‐associated biomarker identification. Identification of infection‐associated markers including ethyl lactate and 3,5‐dimethyloctane informed the development of a high‐sensitivity sensor array integrated into an individually ventilated cage (IVC) platform. Operating at 1 Hz, the system generated 3,628,800 longitudinal measurements over a 7‐day infection cycle, capturing distinct odor‐response dynamics between infected and uninfected groups while exploratory principal component analysis supported stage‐associated temporal patterns. Machine‐learning models, including KNN, SVM, and LDA, leveraged these temporal dynamics to model the complete infection cycle with the best internal accuracy reaching 99.88% and model generalizability further supported by an independent blinded cohort. Notably, early sensor‐response shifts were observed at 7–8 hours post‐infection (hpi), and robust model discrimination was achieved by 9 hpi, supporting the feasibility of early‐stage infection monitoring in this murine model. By fusing biomarker‐guided odor sensing with machine learning, the IOMS enables autonomous, continuous surveillance and highlights the potential of longitudinal VOC monitoring for preclinical respiratory infection studies.

## Graphical abstract

An intelligent odor monitoring system integrates an IVC animal model, gas sensor array, real‐time resistance readout, and machine‐learning analysis to continuously monitor infection‐associated odor changes. The platform captures longitudinal sensor responses, distinguishes infected from healthy states, and supports early‐stage respiratory viral infection discrimination in a non‐invasive cage‐based monitoring workflow.

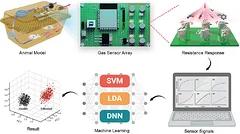

## 1 Introduction

Early diagnosis and real‐time monitoring throughout respiratory viral infection courses are clinically imperative yet technologically unattained. Antiviral efficacy depends critically on timing: oseltamivir, for instance, requires administration within 48 h of influenza symptom onset [1]. However, conventional diagnostics fail to deliver continuous surveillance. Low viral loads during presymptomatic/asymptomatic phases evade detection by RT‐PCR due to insufficient nucleic acid accumulation [2, 3, 4], while late‐stage inflammation post‐viral clearance confounds serological assays [5, 6]. Current gold‐standard methods—including RT‐PCR [7, 8] and rapid tests (e.g., GICA [6], LAMP [9], CRISPR [10])—are inherently single‐point measurements, constrained by: (i) Temporal blind spots (incubation/recovery phases), (ii) Infrastructure dependency (delaying results), (iii) Threshold‐limited sensitivity (early/late stages). This diagnostic void underscores an urgent need for non‐invasive, real‐time technologies capable of continuous viral infection tracking from presymptomatic onset through convalescence. The integration of such monitoring platforms with intelligent diagnostic frameworks represents a groundbreaking innovation, enabling the automation of large‐scale data analysis and remote clinical decision‐making, which are pivotal for advancing precision medicine [11].

Systemically derived volatile organic compounds (VOCs)—direct metabolic byproducts of host‐pathogen interactions and infection‐induced inflammatory cascades—offer a crucial, non‐invasive window into the dynamic processes of respiratory viral pathogenesis when captured in exhaled breath [12, 13]. Species such as decane, phenol, and 2,9‐dimethylundecane have demonstrated diagnostic value for non‐infectious respiratory disorders like asthma [14, 15, 16]. However, acute viral infections present unique diagnostic challenges: their associated VOCs undergo continuous dynamic evolution across latent, acute, and convalescent phases [17], generating transient, multi‐component VOC signatures that demand high temporal‐resolution monitoring [18, 19].

Gas chromatography/mass spectrometry (GC/MS) remains the established gold standard for exhaled VOC biomarker discovery [20]. Yet its high cost, operational complexity, and inability for real‑time analysis limit practical clinical deployment. Artificial olfactory systems, such as electronic noses combining sensor arrays with machine learning, offer a promising alternative by enabling rapid, portable, and cost‑effective VOC profiling [21, 22]. Advanced intelligent gas‐sensing platforms are now being utilized to achieve rapid fingerprinting of volatile metabolites for the point‐of‐care screening of various metabolic and infectious disorders, often surpassing the efficiency of traditional laboratory‐based metrics [23]. Their application in disease diagnostics has attracted growing interest [24, 25, 26, 27]. For example, Liu et al. developed a bioinspired DNA‑MXene sensor array that distinguished gastric, lung, and colorectal cancers from healthy controls through exhaled breath analysis [28]. Similarly, Capuano et al. reported a porphyrin‑based resistive array achieving 93.3% true positive and 86.7% true negative rates for chronic kidney disease detection via linear discriminant analysis [29]. Nevertheless, existing systems remain constrained to discrete‐timepoint or short‐duration VOC analysis due to unresolved challenges in maintaining sensor stability during longitudinal monitoring, suppressing environmental noise in dynamic settings, and establishing standardized calibration protocols for evolving VOC biomarker profiles, fundamental barriers to clinical deployment of continuous infection staging platforms.

To address these limitations, we ‐introduce the Intelligent Odor Monitoring System (IOMS) that is an end‐to‐end platform enabling real‐time, non‐invasive monitoring of respiratory viral infection progression. Diverging fundamentally from conventional electronic noses that analyze static odor samples in controlled environments, IOMS integrates miniaturized multi‐sensor arrays with high‐efficiency particulate air (HEPA) filtration and embedded electronics within an IVC platform, enabling continuous capture of dynamic VOC profiles during natural respiration and systemic emissions—directly addressing the temporal resolution deficit. Crucially, by coupling high‐throughput data acquisition with optimized machine learning models, the system could cover the footprint of VOCs in a full infection cycle. Consequently, IOMS facilitates real‐time VOC biomarker decoupling and dynamic signature learning, with robust performance validated in blinded cohorts, offering a novel breath‐based diagnostic tool.

## 2 Results and Discussion

### 2.1 Design and Characterization of the IOMS Based on Infection‐Associated Biomarkers

**FIGURE 1:**
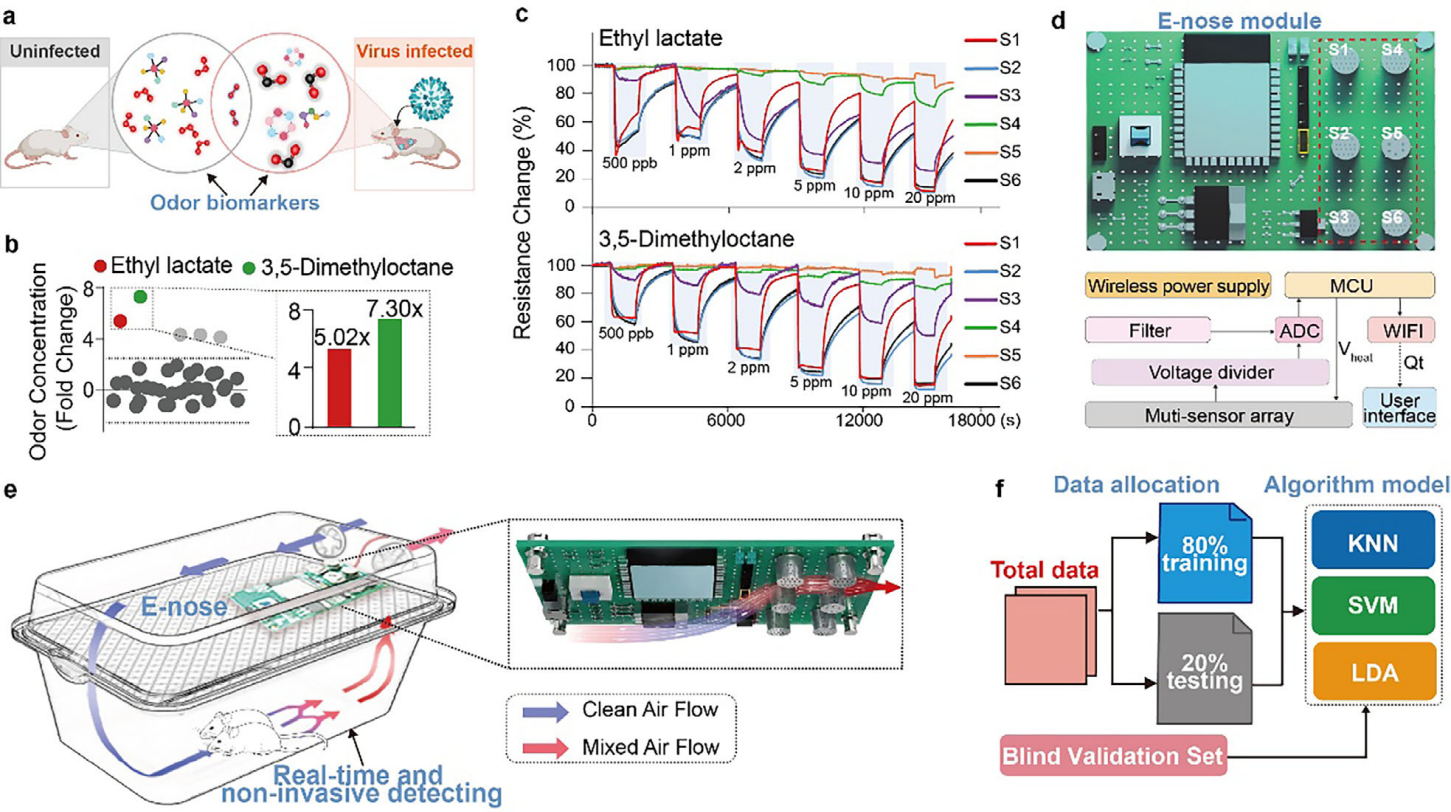
Design architecture and diagnostic workflow of the IOMS for real‐time and non‐invasive viral infection surveillance. (a) Schematic representation of infection‐associated VOC emission from mice during respiratory viral pathogenesis. (b) GC‐MS‐based identification of differentially changed VOCs, highlighting ethyl lactate and 3,5‐dimethyloctane, which were prioritized for downstream sensor‐array design based on their marked abundance changes and suitability for sensor calibration. (c) Sensitivity validation of the six‐sensor array (S1–S6) toward the identified biomarkers across a range of concentrations (500 ppb to 20 ppm). (d) Hardware architecture of the IOMS electronic nose module, including the multi‐sensor array, MCU‐controlled signal processing, and Wi‐Fi transmission units. (e) Integration of the IOMS platform into an individually ventilated cage (IVC) for real‐time and non‐invasive detection under controlled airflow. Filtered air enters through the intake port, mixes with host‐derived VOCs in the cage headspace, passes across the sensor module, and exits through the outlet port under controlled airflow. (f) Analytical workflow for model development and validation. Data from three independent infection experiments were collected. The first two experiments were used as the model‐development cohort and were further divided into training (80%) and internal testing (20%) subsets. Five‐fold stratified cross‐validation was performed during the separation of the training and test sets for parameter optimization and feature selection. The third independent experiment was reserved as an independent blinded validation cohort.

Infected mice exhibited infection‐associated VOC alterations over the course of infection (Figure 1a). To elucidate the chemical basis of these alterations, we first employed headspace solid‐phase microextraction (HS‐SPME) coupled with GC‐MS to analyze the exhaled odor profiles. VOCs were sampled starting at 24 h post‐infection (1 dpi) and additionally at 3, 5, and 7 dpi (days post‐infection), with two biological replicates per time point. Our analysis revealed that several VOC concentrations changed significantly post‐infection, with ethyl lactate and 3,5‐dimethyloctane identified as dominant biomarkers, exhibiting 5.02‐ and 7.30‐fold increases, respectively, at 1 dpi, with these changes persisting through 3 dpi and declining by 5 dpi, partially reflecting the progression of infection. (Figure 1b and Figure S1). These two VOCs were selected as representative targets for sensor‐array optimization based on their marked abundance changes, reproducible detection, and suitability for sensor calibration. To achieve real‐time monitoring of these trace metabolites, we developed the IOMS, which integrates a highly sensitive six‐element semiconductor gas sensor array (S1–S6). Each sensor within the array was validated to exhibit distinct, concentration‐dependent resistance response patterns toward the identified biomarkers, ensuring robust cross‐sensitivity for complex mixture recognition (Figure 1c and Table S1 and Figure S2).

The core of this platform lies in its hardware architecture and seamless integration with the individually ventilated cage (IVC) system. The IOMS electronic nose module utilizes a microcontroller unit (MCU) for precise sensor temperature regulation, instrumentation amplification for signal enhancement, and 16‐bit analog‐to‐digital conversion (ADC) for high‐fidelity acquisition, with data transmitted via Wi‐Fi (Figure 1d). By embedding this module directly within the IVC environment, the IOMS enables the continuous capture of infection‐induced VOC profiles from the cage headspace during natural respiration. The sensor module was mounted inside the cage top cover above the cage bottom, with all six sensor sampling ports oriented toward the animal activity area and positioned along the exhaust airflow pathway, thereby enabling active sampling of exhaled gases from the mice (Figure 1e and Figure S3). This integrated setup leverages the existing HEPA‐filtration system and precisely regulated airflow to maintain a stable and ultra‐clean cage atmosphere. Such a controlled micro‐environment was designed to ensure that the dynamic signals captured by the sensor array predominantly reflected host‐associated metabolic alterations while minimizing background environmental noise. All monitoring experiments were conducted in a tightly controlled ABSL‐2 laboratory environment with regulated temperature, humidity, airflow, and light/dark cycle, thereby minimizing potential environmental fluctuations during longitudinal signal acquisition. The environmental setpoints of the ABSL‐2 facility used for IOMS monitoring are summarized in Table S2. In addition, empty‐cage recordings acquired under the same monitoring configuration showed stable baseline signals across most channels after preprocessing, without systematic fluctuations comparable to the infection‐associated patterns observed in animal experiments (Figure S4 and Table S3). Therefore, blank‐subtraction was not applied in the main analyses, as the empty‐cage baseline did not show progressive drift comparable to the group‐level differences observed during infection monitoring.

The dynamic time‐series dataset spanning the entire disease course laid the foundation for machine learning algorithms, with the selection of algorithms driven by the underlying kinetic features [30, 31]. Utilizing this integrated platform, we conducted continuous 7‐day monitoring at a 1 Hz sampling frequency, generating a massive dataset of 3,628,800 longitudinal measurements per experiment. To develop and evaluate the models, we collected data from three independent infection experiments. Data from the first two experiments were used for model development and were further divided into training (80%) and internal testing (20%) subsets. When distinguishing between the training set and the test set, stratified five‐fold cross‐validation was applied for parameter optimization and feature selection, enabling efficient use of the available training data while improving model stability and generalization assessment. Data from the third independent experiment were reserved as an independent blinded validation cohort and were not used for model training or hyperparameter tuning. Three machine‐learning algorithms—k‐Nearest Neighbors (KNN), Support Vector Machine (SVM), and Linear Discriminant Analysis (LDA)—were then implemented for infection identification and stage‐aware classification (Figure 1f).

### 2.2 Full‐Course Odor Signals of IOMS in a Murine Model

**FIGURE 2:**
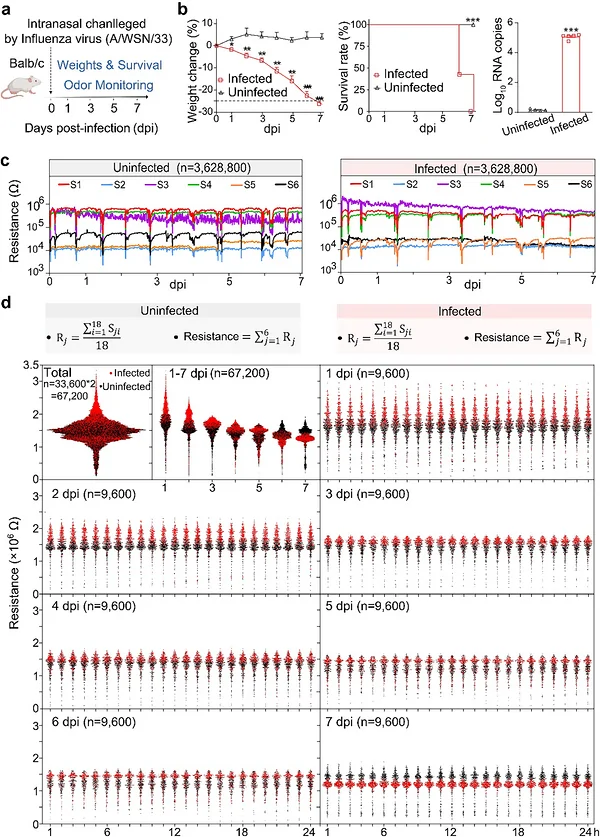
Full‐course odor‐response dynamics and data treatment in the murine model. (a) BALB/c mice were intranasally challenged with 4,000 plaque‐forming units (PFU) (2 LD_50_) of influenza A/WSN/33 (H1N1) virus or PBS (uninfected control). Body weight and survival rate were monitored daily for 7 days, with euthanasia criteria set at ≥ 25% weight loss (*n* = 5 per group). IOMS recorded six real‐time resistance signals at 1 Hz in response to odor changes. (b) Left: Mean body weights (± SD) with dashed line indicating 25% weight loss threshold. Middle: survival curves. Right: Viral *NP* gene copies in lungs quantified by absolute quantitative RT‐PCR (± SD, *n* = 5). (c) Real‐time resistance signals from six highly sensitive gas sensors (S1‐S6) in infected vs. uninfected. (d) Normalized six‐sensor resistance values were summed per measurement and averaged over 18‐second IVC ventilation cycles (33,600 data points per group). The overall resistance values for both uninfected and infected groups over the 7‐day period are shown first. Next, the daily resistance values from 1 to 7 dpi are shown. Finally, hourly resistance fluctuations within each day are presented, with 9,600 data points per day, highlighting the temporal variation in resistance across the 24 h cycle. Each plotted point represents one preprocessed longitudinal measurement rather than a biologically independent replicate. Statistical analyses were conducted using two‐way ANOVA for body weight, log‐rank test for survival analysis, and unpaired *t*‐tests for each experiment (^*^ *p* < 0.05, ^**^ *p* < 0.01, ^***^ *p* < 0.001).

To investigate the temporal response of IOMS to specific odor release in a full‐infection cycle from the incubation phase to the terminal stage, a lethal influenza murine model was established by intranasally infecting BALB/c mice with influenza virus A/WSN/33 (2 LD_50_ = 4,000 plaque‐forming units (PFU)). We monitored the body weight and survival of the mice daily while collecting real‐time resistance signals every second from IOMS over 7 days (Figure 2a). Daily weight monitoring in the infected group revealed progressive decline until 100% mortality by 7 dpi, while lung viral loads peaked at 1.5 × 10^5^ RNA copies/mL (Figure 2b), confirming infection validity. Decoded from the IOMS, 3,628,800 data points were obtained from each group, respectively. The sensor signal traces showed distinct response patterns between the two groups (Figure 2c). The slight baseline differences at the initial time points in Figure 2c, particularly for sensor S3, are more likely attributable to differences in the initial sensor state or minor cage‐to‐cage/system‐level variation, rather than to very early biological divergence. Signal processing revealed the dynamic complexity underlying infection progression. By summing six‐sensor resistance values and averaging over 18‐second (s) IVC ventilation cycles, we obtained 33,600 preprocessed longitudinal measurements per group after averaging over one complete 18‐s ventilation cycle. This window was chosen because it corresponds to one complete IVC ventilation cycle and reduces within‐cycle fluctuations. Temporal analysis uncovered critical phase‐dependent patterns: infected mice showed elevated resistance values versus controls during early phases (1‐3 dpi), but comparable or reduced values during late phases (5‐7 dpi) (Figure 2d and Figure S5). This biphasic response pattern reflects the evolving metabolic landscape of viral pathogenesis—initial inflammatory activation followed by metabolic exhaustion—consistent with infection‐associated metabolic dynamics for our detection approach. These findings confirm that IOMS successfully captures infection‐associated VOC dynamics.

### 2.3 Exploratory PCA of Infection‐Associated Odor‐response Patterns

**FIGURE 3:**
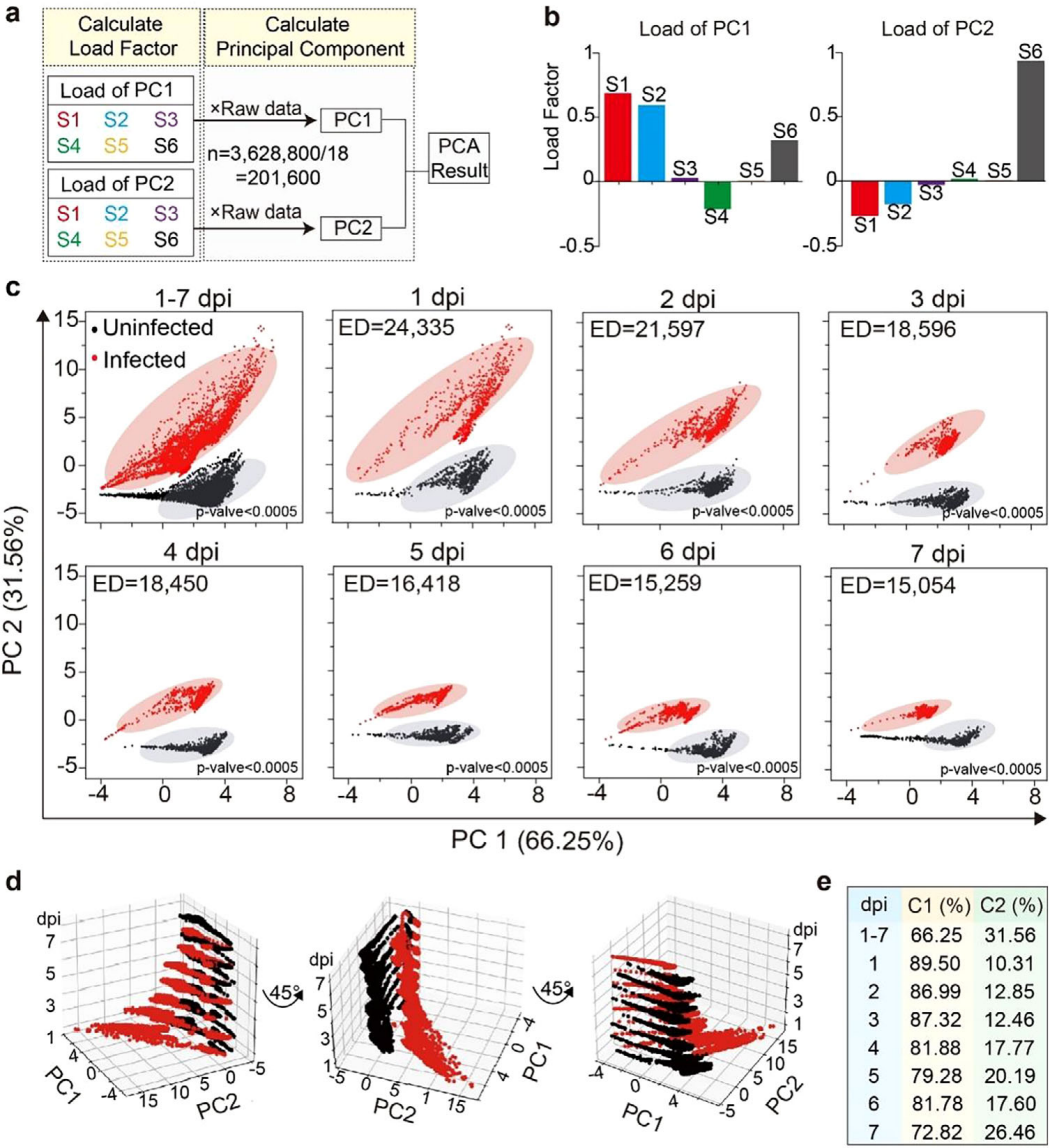
Exploratory principal component analysis of infection‐associated odor‐response patterns. (a) Principal component loadings for six sensors (S1‐S6) in PC1 and PC2 were calculated via Singular Value Decomposition, projecting the six‐dimensional resistance signals to two principal components, PC1 and PC2. These loadings were then applied to the original data to derive PCA results. (b) Principal component loadings for sensors S1‐S6 in PC1 and PC2. (c) PCA visualization revealing distinct clustering of infected (red) and uninfected (black) samples based on odor resistance signals acquired by IOMS. (d) 3D dynamic statistical plot of PCA analysis, with the Z‐axis representing infection time. (e) Variance contribution of the principal components at different stages of infection. PCA was used here as an exploratory dimensionality‐reduction and visualization approach for the longitudinal sensor dataset.

To further distinguish odor signals between infected and uninfected groups, we performed exploratory principal component analysis (PCA) to visualize major variance patterns in the preprocessed sensor‐response data. PCA served to project the six‐dimensional sensor data into two principal components through singular value decomposition (SVD). Loading analysis revealed that sensors S1 (0.694), S2 (0.602), and S6 (0.328) contribute substantially to PC1, while PC2 is primarily influenced by S6 (0.942), with other sensors contributing <0.28 (Figure 3a,b). This differential sensor contribution pattern indicates successful capture of infection‐associated odor‐response patterns through our multi‐sensor array design.

Spatial visualization demonstrated clear separation in PCA space between infected (red) and uninfected (black) clusters in PC1‐PC2 space, with Euclidean distances (ED) quantifying intergroup divergence across the full infection course and daily datasets (Figure 3c). Maximum divergence occurred during early infection phases (1 dpi, ED = 24,335), progressively decreasing toward late stages (7 dpi, ED = 15,054), confirming that early‐phase sensor data inherently contain infection‐characteristic features. Three‐dimensional spatiotemporal analysis using infection timing as the Z‐axis further revealed specific temporal drift patterns unique to infected clusters (Figure 3d). Variance contribution analysis showed PC1 and PC2 explained 66.25% and 31.56% of total variance for the overall datasets (1‐7 dpi), respectively, achieving 97.81% cumulative contribution (Figure 3e). Even higher PC1 contributions were observed in day‐by‐day separate analyses, validating the reliability of our dimensionality reduction approach. These PCA results support the presence of separable infection‐associated odor‐response patterns and can be effectively extracted by our preprocessing pipeline, establishing a robust quantitative framework for subsequent machine learning analysis. PCA is used here as an exploratory visualization tool rather than definitive proof of infection specificity.

### 2.4 Viral Infection Identification Driven by Machine Learning

**FIGURE 4:**
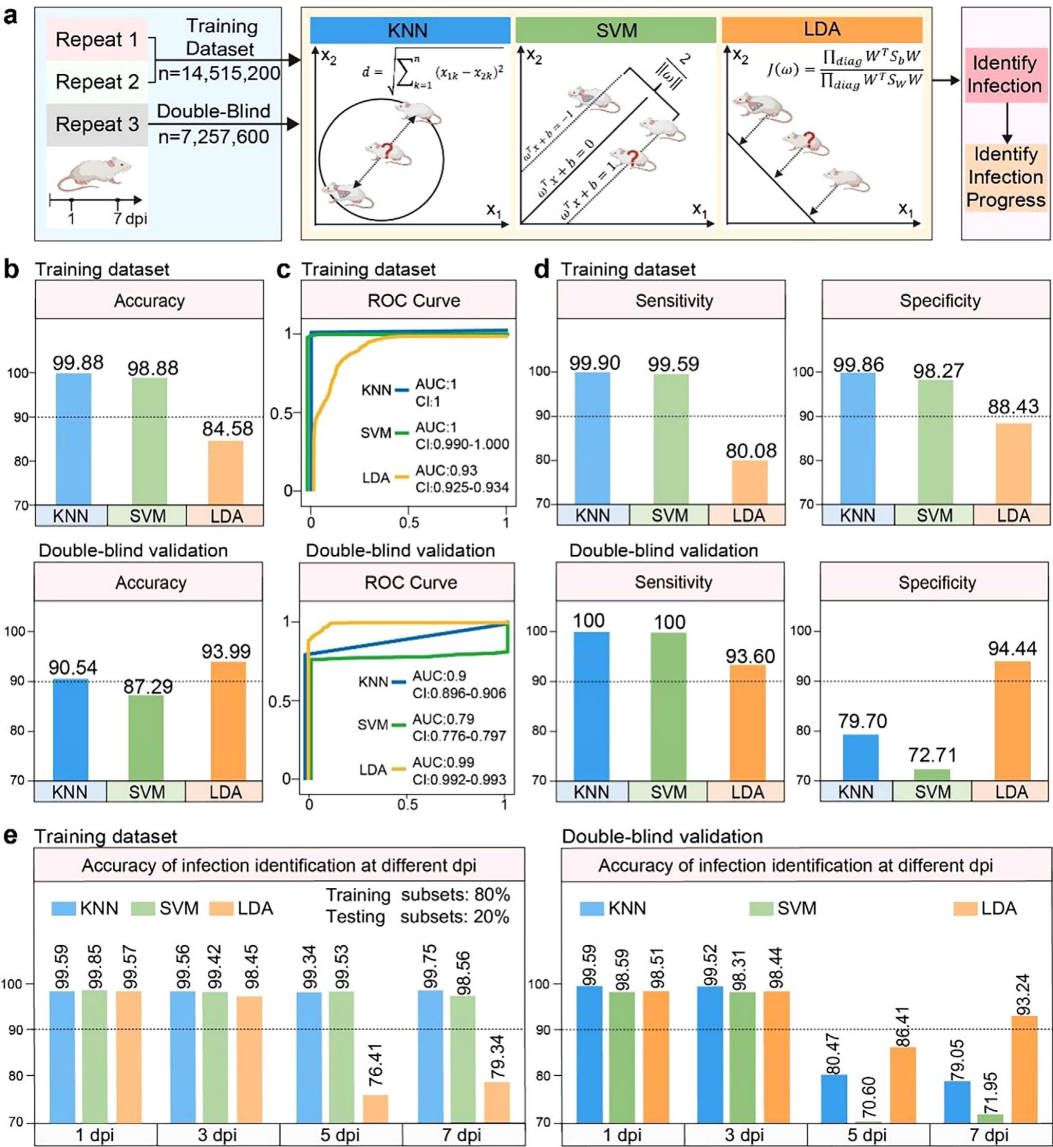
Viral infection identification driven by machine learning. (a) Analytical workflow for model development and validation. Data from three independent mouse infection experiments were collected. Measurements from the first two experiments were used as the model‐development cohort, with an 8:2 split into training and internal testing subsets. Five‐fold stratified cross‐validation was performed during the separation of the training and test sets for parameter optimization and feature selection. Measurements from the third independent experiment were reserved as an independent blinded validation cohort. (b) Classification performance of KNN, SVM, and LDA algorithms in internal evaluation (top) and independent blinded validation (bottom) datasets. (c) The area under the ROC curve indicates strong discriminative ability of the models. (d) Sensitivity and specificity values from the confusion matrix were used to evaluate classification performance. (e) Daily diagnostic accuracies of three machine learning algorithms.

To evaluate the applicability of the IOMS for viral infection detection, we applied machine learning algorithms to extract infection‐associated features from the preprocessed six‐sensor resistance dataset. In clinical tasks, the complexity of data sources and the diversity of disease diagnoses necessitate the use of a range of algorithms to address the challenges posed by such intricate conditions. Data from three independent experiments (n = 21,772,800 total measurements, 3,628,800 longitudinal measurements per experiment for uninfected or infected group) were aggregated to minimize individual variability and batch effects. Two experimental datasets (n = 14,515,200) formed the model‐development cohort with an 8:2 training/testing partition, while the third dataset (n = 7,257,600) served as an independent blinded validation cohort to assess generalization capacity (Figure 4a). Within the development cohort, five‐fold cross‐validation was performed for parameter optimization and feature selection, and the blinded cohort was reserved exclusively for external validation without involvement in model training or tuning.

Algorithm‐specific performance patterns revealed the inherent complexity of dynamic VOC analysis. For infection classification, KNN and SVM achieved exceptional internal testing accuracies of 99.88% and 98.88% respectively on day 7 post‐infection (Figure 4b). However, LDA showed lower internal test accuracy (84.58%) but superior generalization in blinded validation (93.99%), significantly outperforming KNN (90.54%) and SVM (87.29%). This performance divergence across identical datasets demonstrates that algorithm selection can match specific data characteristics and intended application scenarios. ROC analysis confirmed robust discriminative ability with AUC values consistently exceeding 0.8 for both internal evaluation and blinded validation (Figure 4c). Confusion matrix analysis revealed sensitivity values >98% for KNN and SVM algorithms, with LDA achieving >90% sensitivity and specificity in blinded validation (Figure 4d). These complementary performance profiles highlight the need for algorithm optimization based on deployment requirements.

Although longitudinal disease monitoring presents greater challenges than infection diagnosis due to increased temporal variability and data complexity, the developed algorithms remain applicable for assessing infection status across various stages of disease progression. All algorithms achieved >98% classification accuracies during the first three days post‐infection in both internal evaluation and blinded validation (Figure 4e). The ability to maintain high accuracy across different disease stages while using task‐specific algorithmic strengths demonstrates the power of our comprehensive approach. The complementary strengths of these algorithms will facilitate the efficient deployment of IOMS for future continuous infection‐monitoring applications.

### 2.5 Ultra‐Early Infection Detection Using IOMS

**FIGURE 5:**
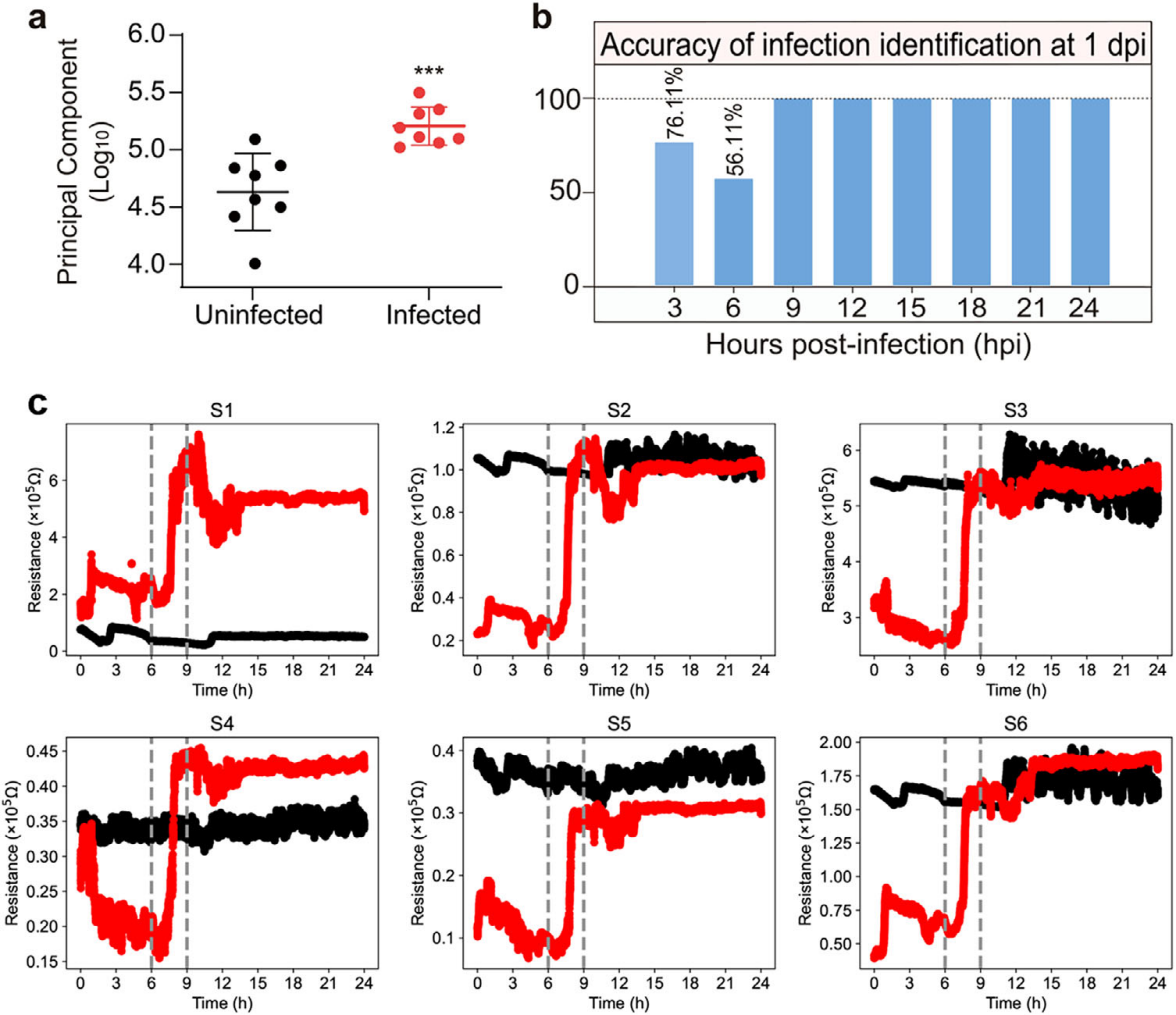
Early‐stage detection performance of IOMS during the first day of infection. (a) Exploratory PCA of preprocessed sensor‐response patterns for every 3 hours within 1 dpi, showing separation between infected and control groups in principal‐component space. Each point represents one preprocessed longitudinal measurement obtained after averaging over a complete 18‐s ventilation cycle. (b) Classification performance for infection identification at 3‐h intervals during the first 24 hpi. The plot shows the temporal evolution of model performance, with robust discrimination achieved by 9 hpi. (c) Real‐time resistance dynamics of the six sensors (S1–S6) between 0 and 24 hpi in the infected and control groups. A coordinated increase in resistance was observed in the infected group between 7 and 8 hpi relative to the comparatively stable control group, indicating an early infection‐associated shift in odor‐response dynamics. The experiment were conducted in cages containing n = 5 mice per group. Data are presented as mean ± SD. Statistical analyses were conducted using unpaired *t*‐tests for each experiment (^*^ *p* < 0.05, ^**^ *p* < 0.01, ^***^ *p* < 0.001).

Previous experimental results demonstrated the outstanding performance of the IOMS in early infection detection. To further investigate this capability, we performed exploratory PCA on the preprocessed sensor signals from the infected and control groups at 1 dpi, which revealed a clear separation between the two cohorts. By averaging the principal component scores, we observed distinct clusters for each group, indicating that infection‐associated odor‐response differences were detectable at this stage (Figure 5a). Building on these findings, we conducted a granular temporal analysis to evaluate infection identification during the critical first 24 h post‐infection (hpi). Machine learning results revealed that while initial detection was possible, the IOMS achieved robust discrimination as early as 9 hpi (Figure 5b). These findings further support the feasibility of early‐stage infection monitoring with IOMS in this murine model.

To explore the underlying cause of the rapid increase in diagnostic accuracy observed during the 6–9 hpi period, we analyzed the real‐time resistance dynamics of the six sensors. As shown in Figure 5c, the resistance values of the infected group increased sharply between 7 and 8 hpi, with each sensor exhibiting approximately a threefold rise compared to the comparatively stable 1–6 hpi baseline; during the remaining time periods, only routine fluctuations were observed. The control group exhibited only routine fluctuations throughout 0–24 hpi. This synchronized signal surge may reflect infection‐associated metabolic changes detectable in the cage headspace, although additional time‐resolved biological validation will be required to define its precise causal basis. Taken together, these findings indicate that the IOMS can accurately capture early dynamic changes in infection‐associated odor signals, supporting its potential application for real‐time, early‐stage infection monitoring.

## 3 Discussion

Compared with representative breath‐based electronic‐nose systems reported for disease monitoring, IOMS is distinguished less by sensor number than by its biomarker‐guided design and continuous longitudinal monitoring format. A summary comparison of representative systems is provided in Table S4. Existing electronic‐nose platforms have been applied to a wide range of disease settings, including lung cancer, COPD, COVID‐19, respiratory tract infection, chronic kidney disease, cancer recognition, and metabolic screening, but most of these studies were based on point‐in‐time breath classification rather than dynamic longitudinal monitoring. In contrast, IOMS was designed for biomarker‐informed, 1‐Hz continuous monitoring across the full 7‐day infection course in a controlled IVC setting and further incorporated an independent blinded validation cohort. These features position IOMS as a platform for dynamic infection tracking and stage‐aware monitoring, rather than only endpoint classification.

The present study has several limitations. First, this work represents a proof‐of‐concept demonstration performed under tightly controlled ABSL‐2 conditions with regulated temperature, humidity, airflow, and light/dark cycles; therefore, further studies will be required to evaluate the robustness and applicability of the IOMS in more complex and variable real‐world environments. Second, the IOMS measures infection‐associated VOCs in the cage headspace rather than purified exhaled breath from individual animals, and thus captures a composite signal arising from respiration, body emissions, and cage‐level metabolic activity. Future studies should perform more systematic background VOC profiling and compare VOC distributions across empty, control, and infected cages to further strengthen the causal link between infection‐associated odor‐response patterns and infection‐specific metabolic dynamics across different stages of the infection cycle. Notably, periodic resistance fluctuations were observed in both infected and control groups, which are more likely attributable to endogenous circadian‐driven physiological behavior than to environmental interference or sensor instability. Consistent with this interpretation, group‐housed mice exhibit characteristic circadian activity peaks at the onset and toward the offset of the dark phase, while influenza infection primarily reduces overall activity without disrupting the underlying rhythmic structure. In addition, although coordinated signal changes were consistently observed at 7–8 hpi, the biological basis of this early response still requires further validation through time‐resolved GC‐MS measurements and viral kinetic analyses. Finally, occasional drift‐like fluctuations may occur during long‐term continuous monitoring, as observed for sensor S5 around day 4; however, these fluctuations did not affect downstream analyses after preprocessing and normalization. Future improvements of the IOMS will focus on both system‐level optimization and selective sensing‐material design strategies, including defect‐mediated specific catalysis and orbital‐level chemical coding, to further enhance gas discrimination in complex VOC environments [32, 33].

## 4 Conclusion

The IOMS provides a platform for non‐invasive and real‐time monitoring of respiratory viral infection in murine models, addressing a key limitation of conventional electronic noses that are typically restricted to discrete time‐point analysis. Based on identified infection‐associated biomarkers such as ethyl lactate and 3,5‐dimethyloctane, we developed this biomarker‐driven platform by integrating an IVC‐embedded multi‐sensor array with machine‐learning analysis. Our system enables continuous capture of infection‐associated odor‐response dynamics at second‐scale resolution over a 7‐day infection course. Using a longitudinal murine dataset spanning the full course of infection, IOMS achieved high infection‐identification performance and robust early‐stage discrimination by 9 hpi in this model. Collectively, IOMS serves as an experimental platform for dynamic infection monitoring and provides a framework for investigating host‐pathogen metabolic interactions in preclinical settings.

## 5 Experimental Section

### 5.1 Solid‐Phase Microextraction‐Gas Chromatography‐Mass Spectrometry

VOCs were collected from the IVC cage headspace using HS‐SPME at 1, 3, 5, and 7 dpi. The VOC sampling experiment was performed with two cage‐level biological replicates for each group at each time point, with five mice housed in each IVC cage. Because VOCs were collected from the cage headspace rather than from individual mice, each cage was considered one biological sampling unit. The VOC data presented in the main text represent the mean values of the two cage‐level biological replicates, which showed consistent temporal trends. Samples were incubated with preconditioned SPME fibers (57318, Supelco; aged at 270°C for 30 min) to remove potential interfering artifacts. The fibers were exposed to the IVC cage headspace for 24 h using specific holders (57330‐U, Supelco), followed by thermal desorption at 250°C for 5 min in the GC injector. GC‐MS analysis was performed on a Varian 450GC‐320MS system equipped with a DB‐5 capillary column (30 m × 0.25 mm × 0.25 µm). Helium (99.999%) was used as the carrier gas at a constant flow rate of 1.2 mL/min. The oven temperature program was initiated at 40°C (held for 2 min), ramped to 250°C at 10°C/min, and maintained at 250°C for 5 min. Mass spectra were acquired in electron ionization (EI) mode at 70 eV with a full scan range of m/z 35–500. Compound identification was performed using a multi‐criteria approach to ensure confidence. First, the retention indices (RI) of all detected peaks were calculated using a homologous series of C7–C40 n‐alkanes (Sigma‐Aldrich) analyzed under the same conditions. Putative identification was achieved by matching mass spectra with the NIST11 library (similarity threshold > 85%) and filtering results where the experimental RI matched the library RI (within a ± 20 tolerance). Crucially, the identities of potential biomarkers were further confirmed by comparing their retention times and mass fragmentation patterns with those of authentic standards. Quantitative analysis was performed using 1 ppm deuterated toluene as an internal standard, and relative concentrations were determined based on the peak area ratio. Ethyl lactate and 3,5‐dimethyloctane were prioritized for downstream sensor‐array design due to reproducible and pronounced abundance changes at 1 dpi (fold changes 5.02 and 7.30) that persisted through 3 dpi and decreased by 5 dpi, reflecting disease progression. Other VOCs were excluded due to limited reproducibility or single‐day changes.

### 5.2 Murine Model of Influenza Virus Infection

The influenza A/WSN/33 (H1N1) virus was rescued via reverse genetics as previously described [34] and propagated in Madin‐Darby canine kidney (MDCK) cells. Eight‐week‐old female BALB/c mice (Beijing Vital River Laboratory Animal Technology Co., Ltd.) were randomized into infection (*n* = 5, receiving 50 µL intranasal inoculation of 4,000 plaque‐forming units (PFU) A/WSN/33 under isoflurane anesthesia) or control (*n* = 5, receiving PBS) groups. Each independent experiment included one infected cage and one control cage, and each cage was monitored with an independent IOMS unit throughout the observation period. Mice were housed in IVC and were under identical environmental conditions (22 ± 1°C, 55 ± 5% humidity, 12‐h light/dark cycle). Body weight was monitored daily, and euthanasia criteria included ≥ 25% weight loss or severe morbidity. Real‐time resistance signals from the six‐sensor IOMS array (S1–S6) were continuously recorded at 1 Hz throughout the 7‐day observation period. Three independent infection experiments were performed, and one complete experiment was reserved as an independent blinded validation cohort for model generalization analysis. All infections were conducted in the ABSL‐2 facility at Wuhan University and approved by the Institutional Animal Management and Use Committee (IACUC, No. SKLV‐AE2022‐009).

### 5.3 Quantitative Reverse Transcription Polymerase Chain Reaction (qRT‐PCR)

Viral load in lung tissue was quantified via absolute qRT‐PCR. Initially, 200 µl lung tissue homogenates (NZK‐802DS homogenizer, NovaStar) underwent RNA extraction with TRIzol reagent (15596018, Invitrogen). RT was subsequently performed using oligo(dT) and random primers (RR037A, Takara). A standard curve (10^2^–10^8^ copies/µL) was generated using the pCMV‐IAV‐NP plasmid. qPCR was performed on a 384‐well plate (4343814, Thermo Fisher Scientific) with *NP*‐specific primers (Forward: 5'‐AACGGCTGGTCTGACTCACATGAT‐3', Reverse: 5'‐AGTGAGCACATCCTGGGATCCATT‐3') at 95°C for 10 min, followed by 40 cycles of 95°C for 15 s and 60°C for 1 min. Triplicate reactions were analyzed with copy numbers derived from the standard curve (*R^2^* > 0.99).

### 5.4 Construction of the IOMS and Data Analysis

The IOMS comprises two main components: an electronic nose module and an IVC (Figure 1d,e and Figure S3). The electronic nose is equipped with a gas sensor array, along with data acquisition, processing, and transmission modules (Figure 1d and Figure S3 c,d). The sensor array integrates six commercial gas sensors (S1–S6) (Table S1), each exhibiting distinct resistance‐based responses to different gases. A sampling circuit collects the circuit voltage altered by these resistance changes using a series voltage division principle. The voltage values are recorded by an ADS114S08 chip, transmitted to a Wi‐Fi‐connected computer, and subsequently used to compute the corresponding resistance data in real‐time. The IVC is connected to a HEPA‐filtration system, allowing precise control over airflow rate and ventilation intervals (18 s per cycle). Filtered air entered the cage through the intake port, mixed with host‐derived VOCs in the cage headspace, passed across the electronic‐nose module, and exited through the outlet port. In this configuration, the sensor module continuously sampled the cage‐headspace odor stream during normal animal respiration and routine cage ventilation. The controlled airflow path was designed to reduce environmental particulate interference and to improve the stability of longitudinal signal acquisition. For downstream analysis, raw 1‐Hz resistance signals from all six sensors were preprocessed by averaging over each complete 18‐s ventilation cycle. This preprocessing step was chosen because it matched the fixed gas‐exchange period of the IVC system and reduced within‐cycle fluctuations in the raw sensor traces. The resulting longitudinal measurements were then used for visualization and machine‐learning analyses. To ensure accurate monitoring of metabolic outputs, all mice were maintained in IVC under uniform environmental conditions, including a temperature of 22 ± 1°C, 55 ± 5% humidity, and regulated airflow. To assess baseline system stability, empty‐cage recordings were collected under the same airflow and environmental settings and processed using the same 18‐s averaging pipeline as the animal data. Summary stability analyses are provided in (Table S3).

### 5.5 Principal Component Analysis

PCA was employed to visualize odor‐resistance signal divergence between infected and uninfected groups, encompassing daily monitoring and full‐course comparisons (1‐7 dpi). The analytical workflow proceeded as follows: First, resistance values from six sensors (S1–S6) were normalized by calculating 18‐s interval averages, generating a standardized dataset. Subsequently, the covariance matrix was computed from this dataset, followed by the derivation of eigenvectors and eigenvalues. Eigenvectors defined principal component axes (PC1 and PC2), while eigenvalues quantified the variance explained by each component. Following eigenvalue ranking, dominant eigenvectors were selected to construct the principal component matrix. Finally, matrix projection was achieved by multiplying the standardized dataset with the principal component matrix, yielding PCA score plots. Infected and uninfected groups formed distinct clusters in PCA space. Inter‐group divergence was quantified via mean Euclidean distance between all datapoints across cohorts, providing a metric for systemic odor‐resistance signal differences.

### 5.6 Pattern Recognition Algorithms

Resistance values from the six sensors (S1–S6) were used as six‐dimensional features to construct a six‐dimensional sensor dataset (S1–S6), which was subsequently input into machine learning models. Three classification algorithms—Linear Discriminant Analysis (LDA), Support Vector Machine (SVM; radial basis function kernel), and k‐Nearest Neighbors (KNN; k = 5)—were implemented for model development and evaluation. Data were collected from three independent infection experiments. The first two experiments constituted the model‐development cohort, whereas the third experiment was reserved as an independent blinded validation cohort. Within the model‐development cohort, the dataset was divided into training (80%) and internal testing (20%) subsets. The internal testing subset was used only for model evaluation and did not participate in model fitting, parameter optimization, or feature selection. Stratified five‐fold cross‐validation was applied during model development to optimize model parameters and perform feature selection. This strategy enabled efficient use of the available training data without requiring additional experiments and allowed model stability and generalization to be assessed during development. After model optimization, the final model was evaluated on the internal testing subset and subsequently assessed on the independent blinded validation cohort to determine its external generalization performance. Because the sensor dataset consisted of densely sampled longitudinal measurements, these observations were treated as high‐density time‐series measurements for model development rather than as biologically independent replicates.

### 5.7 Statistical Analysis

Statistical analysis was conducted using GraphPad Prism 9 (GraphPad Software, USA). PCA and machine‐learning analyses were implemented in Python 3.9 using PyTorch. Raw sensor resistance signals from both infected and non‐infected groups were first normalized using min‐max scaling to map the resistance of each sensor to the range of 0–1, thereby minimizing inter‐group baseline differences while preserving within‐group temporal response patterns. The normalized signals were then averaged over each complete 18‐s ventilation cycle to generate preprocessed longitudinal measurements for downstream analysis. No manual exclusion of data points was performed unless otherwise stated. Data are presented as mean ± standard deviation (SD) unless otherwise indicated. The sample size (n) for each experiment is provided in the corresponding figure legend. Statistical analyses for Figure 2b were conducted using two‐way ANOVA for body weight, log‐rank test for survival analysis, and unpaired t‐tests for viral load comparisons. Statistical significance was set at ^*^ for *p* < 0.05, ^**^ for *p* < 0.01, and ^***^ for *p* < 0.001. PCA was used as an exploratory dimensionality‐reduction and visualization approach for the preprocessed longitudinal sensor dataset. Machine‐learning model performance was evaluated using an 8:2 split into training and internal testing subsets within the model‐development cohort, with five‐fold cross‐validation performed during model development for parameter optimization and feature selection, and model generalizability assessed using an independent blinded validation cohort. Because the sensor dataset consisted of densely sampled longitudinal measurements, these observations were interpreted as high‐density time‐series measurements rather than biologically independent replicates.

## Author Contributions

H. L. and K.X. conceived the project and designed the experiments. HY.L., L.L., L.G., and W.Z. designed the IOMS sensor array, completed the IOMS assembly including modifying the mouse cage, configuring wireless connectivity, and installing wireless charging devices. Y.S., YC.Z., W.Y., and Z.Z. conducted influenza virus intranasal infection experiments in mice and collected daily resistance signals acquired by IOMS. BZ.Y. and K.L. performed PCA on IOMS‐collected data. L.L., K.L., and Z.L. conducted data grouping, training/testing of different machine learning algorithm models, and double‐blind experiments with IOMS data. L.L., K.L., and Y.D. calibrated the IOMS sensor array using standard gases. Y.S., YC.Z., W.Y., and B.Y. employed SPME to collect exhaled odors from infected and uninfected mice, followed by gas composition analysis and data processing using GC‐MS systems. K.X., H. L., Y.S., W.Y., L.L., HY.L., HG.L., YL.Z., YC.Z., SM.K., and K.L. wrote the manuscript with input from all the other authors. All authors approved the final manuscript.

## Conflicts of Interest

The authors declare no conflicts of interest.

## Supporting information




**Supporting File**: advs76244‐sup‐0001‐SuppMat.docx.

## Data Availability

The data that support the findings of this study are available from the corresponding author upon reasonable request.
