# Supplementary material for: Real‐Time and Non‐Invasive Detection of Respiratory Viral Infections Using an Intelligent Odor Monitoring System (IOMS)
Source: Adv Sci (Weinh). 2026 Jun 23;13(51):e76244. doi: 10.1002/advs.76244 (PMC13336894; doi:10.1002/advs.76244)
Supplement: Supplementary file 1 [file ADVS-13-e76244-s001.docx]

**Real-Time and Non-Invasive Detection of Respiratory Viral Infections Using an Intelligent Odor Monitoring System (IOMS)**

Yajie Shen^1†^, Weifeng Yuan^1†^, Long Li^2†^, Yucheng Zheng^1^, Kenan Liu^2^, Hegeng Li^2^, Hua-Yao Li^2^, Shu-ming Kuo^1^, Binzhou Ying^2^, Lanpeng Guo^2^, Wenjian Zhang^2^, Zirui Zhang^1^, Yufan Deng^2^, Bohan Yin^1^, Zhaocheng Luo^2^, Ke Xu^1,3*^, Huan Liu^2*^

^1^State Key Laboratory of Virology and Biosafety, Institute for Vaccine Research, College of Life Sciences, Wuhan University, Wuhan, Hubei, 430072, P.R. China.

^2^School of Integrated Circuits, Wuhan National Laboratory for Optoelectronics, Optics Valley Laboratory, Huazhong University of Science and Technology, 1037 Luoyu Road, Wuhan 430074, Hubei, P.R. China.

^3^ School of Public Health, Wuhan University, Wuhan 430071, Hubei, P.R. China.

^†^These authors contributed equally: Yajie Shen, Weifeng Yuan, Long Li

^*^Corresponding author Email: Address correspondence and reprint requests to Dr. Huan Liu (E-mail: [huan@hust.edu.cn](mailto:huan@hust.edu.cn), Tel.: 027-87542594) and Dr. Ke Xu (E-mail: [xuke03@whu.edu.cn](mailto:xuke03@whu.edu.cn), Tel.: 86-27-68756997, Fax: 86-27-68754592).

**This PDF file includes:**

Figures. S1 to S5

Table. S1 to S4

**Figure S1. Stage-specific odor dynamics.** Fourteen differentially expressed odors at each infection stage (1, 3, 5, and 7 dpi), comparing to uninfected mice, as depicted by concentration fold change plot and heatmaps, alongside temporal variation profiles for 9 odors exhibiting significant concentration shifts.

**Figure S2. Detection limits and response curves of sensors (S1–S6) for ethyl lactate and 3,5-dimethyloctane.** The detection limits (LoD) for ethyl lactate are 3.85 ppb, 0.71 ppb, 5.45 ppb, 35.47 ppb, 57.99 ppb, and 3.06 ppb for sensors S1-S6, respectively. The detection limits for 3,5-dimethyloctane are 5.31 ppb, 1.43 ppb, 21.15 ppb, 88.93 ppb, 405.48 ppb, and 18.44 ppb for sensors S1-S6, respectively. The response curves of the sensors to both gases follow power-law relationships with gas concentration, in accordance with the law of mass action. The R² values for the fits are indicated in the corresponding plots, demonstrating excellent linearity and sensitivity in the ppb range for both compounds.

**Figure S3. Photograph of the IOMS and hardware module.** (a-b) The sensor array of the IOMS is located inside the IVC. This enclosed environment eliminates external interference, enabling precise monitoring of meteorological conditions within the cage. By accurately controlling factors such as temperature, humidity, and airflow, a controlled experimental setup can be established to accurately simulate the environmental conditions of respiratory infections. (c-d) Multi-sensor array and wireless architecture. The array comprises six commercial odor sensors, wireless charging modules, and real-time data transmission modules for continuous monitoring.

**Figure S4. Resistance signal variations during continuous 7-day monitoring of the empty IOMS system.** Sensors S1-S6 were recorded under the same IVC and ABSL-2 environmental conditions but without animals. The empty-cage baseline remained stable over the 7-day period, with low coefficients of variation for all six sensor channels (Table S3), indicating limited system-level and environmental background fluctuations.

**Figure S5. Temporal evolution of sensor discrimination performance.** Temporal distribution of resistance responses from six sensors (S1–S6) at 1, 3, 5, and 7 days post-infection (dpi), comparing infected (red) and uninfected (blue) groups. The violin plots reflect the distribution and density of resistance values (×10⁵ Ω) over time.

**Table S1| Functional Parameters of sensors** **in the IOMS**

| **Number** | **Sensor model** | **Odor type** | **Primary target VOCs** | **Detection range (ppm)** |
| --- | --- | --- | --- | --- |
| S1 | WSP2110 | Aromatic | benzene, toluene, ethanol | 300~10,000 |
| S2 | MP-2 | Aliphatic | propane | 300~10,000 |
| S3 | MP-3B | Alcohol | ethanol | 200~10,000 |
| S4 | MP-4 | Aliphatic | methane | 1~1,000 |
| S5 | MP-5 | Aliphatic | propane, butane | 0~500 |
| S6 | MP503 | Aldehyde | formaldehyde | 1~50 |

Notes: Sensors were sourced from Hanwei Electronics Co., China. Detection ranges represent minimum detectable concentration (MDC) to saturation threshold under standard conditions (25°C, 1 atm).

**Table S2|** **Environmental setpoints of the ABSL-2 facility used for IOMS monitoring experiments**

| Parameter | Setpoint / Controlled Range | Description |
| --- | --- | --- |
| Temperature | 22 ± 1 °C | Controlled room temperature during animal monitoring |
| Relative Humidity | 55 ± 5% | Controlled room humidity during animal monitoring |
| Light/Dark Cycle | 12 h / 12 h | Standard light/dark cycle maintained in the ABSL-2 facility |
| Airflow | Controlled IVC ventilation | Constant airflow maintained by the IVC system |
| Inlet Air | HEPA-filtered laboratory air | Air supplied to the IVC system after HEPA filtration |
| Monitoring Environment | ABSL-2 facility | All infection-monitoring experiments were conducted under biosafety-controlled laboratory conditions |

Notes: This table summarizes the environmental setpoints of the ABSL-2 facility used for the animal monitoring experiments. These values represent the controlled operating conditions of the facility and do not constitute continuous real-time environmental recordings from the experimental days.

**Table S3| Summary statistics of empty-cage baseline signals across the six sensor channels**

| **Sensor Channel** | **Mean Signal** | **Standard Deviation** | **Coefficient of Variation (%)** | **Variability Level** |
| --- | --- | --- | --- | --- |
| S1 | 299,199.28 | 13,742.64 | 4.59% | Low |
| S2 | 22,730.30 | 634.46 | 2.79% | Low |
| S3 | 1, 530, 031.33 | 26, 749.70 | 1.75% | Low |
| S4 | 630, 075.47 | 16, 995.50 | 2.70% | Low |
| S5 | 44, 242.25 | 2, 284.87 | 5.16% | Low |
| S6 | 70, 442.66 | 909.86 | 1.29% | Low |

Notes: Values were calculated from empty-cage baseline recordings collected under the same airflow and environmental conditions as the animal experiments. Mean signal, standard deviation, and coefficient of variation (CV) were used to assess channel stability after preprocessing. Variability level was categorized based on CV values.

**Table S4| Comparison of IOMS with representative breath-based electronic-nose systems for disease monitoring**

| **Electronic nose systems** | **Number of sensors** | **Sample size** | **Algorithm** | **Accuracy** | **Monitoring / Early detection feature** |
| --- | --- | --- | --- | --- | --- |
| Aeonose (lung cancer, 6 patients) | 3 (metal oxide semiconductor sensors) | Lung cancer: patient group n=6, healthy group n=10 | Artificial neural network (ANN) | Accuracy 93.8%, sensitivity 85.7%, specificity 100% |  |
| FGC eNose (HERACLES II) for COPD | 2 (dual-column fast gas chromatography) | COPD: patient group n=23, healthy group n=336 | Principal component analysis (PCA) | Accuracy 82.2%, sensitivity 96%, specificity 91% |  |
| Gold nanoparticle sensor array (COVID-19) | 8 | COVID-19 patients n=49, non-COVID pulmonary infection group n=33, healthy group n=58 | Quadratic discriminant analysis (QDA), LDA, ROC | COVID-19 vs. healthy: training/test accuracy 94%/76%; COVID-19 vs. non-COVID infection: 90%/95% |  |
| E-nose system with TGS1820, TGS2620, TGS2600, MQ3, NO2, B2S, SGX_NO2, SGX_H2S, K33 CO2, ALN-03P, AL-03S | 11 | 151 participants (92 women, 59 men); age 67 ± 9.3 years; training set 136 patients (272 samples), test set 15 patients (30 samples)* | LGBMRegressor (Light Gradient Boosting Machine) | Entire range: MAPE 13.7%, MAE 21.2 mg/dL, R²=0.22; Normal range (<200 mg/dL): MAPE 8%, MAE 12.9 mg/dL, R²=0.52 | First proposal of noninvasive total cholesterol level measurement using exhaled breath |
| rGO-based e-nose sensor array modified with MOFs (Ni(HITP)2, Cu(HHTP), Co(HITP)2) and MPcs (NiPc, FePc) | 8 / 6 (2 sites with low SNR excluded) | 145 clinical samples (89 bacterial infections + 56 viral infections); independent external validation set of 43 cases (37 classified, 6 unconfirmed) | Weighted fusion classification model (SVM + Random Forest + Lasso regression) | Validation set: 83.7% (AUC=0.87); External test set: 75.7% (AUC=0.81); Mycoplasma infection LDA classification: 75% | Room-temperature rapid noninvasive differentiation of bacterial vs. viral respiratory infections |
| DNA/MXene (Ti3C2) bioinspired biosensor array (6C-BBA) | 6 (Ti3C2, DNA-A/Ti3C2, DNA-T/Ti3C2, DNA-C/Ti3C2, DNA-G/Ti3C2, DNA-AT3/Ti3C2) | 43 volunteers (gastric cancer, lung cancer, colorectal cancer patients + healthy controls); 20 measurements per breath type, total 860 datasets | KNN, SVM, LR, LDA, CART, NB (KNN best performer) | Unmodified 6C-BBA: healthy 81.4%, gastric cancer 63.2%, lung cancer 61.1%, colorectal cancer 69.6%; Chemically modified 6C-BBA: gastric cancer 86.3%, lung cancer 94.1%, colorectal cancer 89.5%, healthy 86.3% | Early noninvasive cancer recognition (gastric, lung, colorectal cancer) |

| **Reference** | **Corresponding Table Entry** | **Verification Link** |
| --- | --- | --- |
| Marzorati, D., et al. A Metal Oxide Gas Sensors Array for Lung Cancer Diagnosis Through Exhaled Breath Analysis. Annu Int Conf IEEE Eng Med Biol Soc. 2019, 1584–1587. | Aeonose (lung cancer, 6 patients) | https://pubmed.ncbi.nlm.nih.gov/31946198/ |
| Rodriguez, A. M., et al. Ultrafast gas chromatography coupled to electronic nose to identify volatile biomarkers in exhaled breath from chronic obstructive pulmonary disease patients: a pilot study. Biomed. Chromatogr. 33, e4684 (2019). | FGC eNose (HERACLES II) for COPD | https://doi.org/10.1002/bmc.4684 |
| Shan, B., et al. Multiplexed nanomaterial-based sensor array for detection of COVID-19 in exhaled breath. ACS Nano 14, 12125–12132 (2020). | Gold nanoparticle sensor array (COVID-19) | https://pubs.acs.org/doi/10.1021/acsnano.0c05657 |
| Paleczek, A., et al. Noninvasive Total Cholesterol Level Measurement Using an E-Nose System and Machine Learning on Exhaled Breath Samples. ACS Sens. 9, 6630–6637 (2024). | E-nose system for cholesterol measurement | https://pubs.acs.org/doi/10.1021/acssensors.4c02198 |
| Xu, S., et al. Discrimination of Respiratory Tract Infections by a Reduced Graphene Oxide Array Modified with Metal-Organic Frameworks and Metal Phthalocyanines. ACS Nano 2025, 19, 20, 19429–19441. | rGO-based e-nose sensor array for respiratory infections | https://pubs.acs.org/doi/10.1021/acsnano.5c04231 |
| Liu, S., et al. DNA-Mediated Bioinspired MXene Gas Sensor Array with Machine Learning for Noninvasive Cancer Recognition. ACS Nano 19, 25363–25384 (2025). | DNA/MXene biosensor array for cancer recognition | https://pubs.acs.org/doi/10.1021/acsnano.5c06893 |
| Capuano, R., et al. Disposable Sensor Array Embedded in Facemasks for the Identification of Chronic Kidney Disease. ACS Sens. 10, 4850–4861 (2025). | Porphyrin-doped PEDOT/PSS array for CKD screening | https://pubs.acs.org/doi/10.1021/acssensors.4c03227 |

Notes: Performance metrics are reported as described in the original studies.
